# Claisened Hexafluoro Inhibits Metastatic Spreading of Amoeboid Melanoma Cells

**DOI:** 10.3390/cancers13143551

**Published:** 2021-07-15

**Authors:** Angela Leo, Erica Pranzini, Laura Pietrovito, Elisa Pardella, Matteo Parri, Paolo Cirri, Gennaro Bruno, Maura Calvani, Silvia Peppicelli, Eugenio Torre, Maiko Sasaki, Lily Yang, Lei Zhu, Paola Chiarugi, Giovanni Raugei, Jack L. Arbiser, Maria Letizia Taddei

**Affiliations:** 1 Department of Experimental and Clinical Biomedical Sciences “Mario Serio”, University of Florence, Viale Morgagni 50, 50134 Florence, Italy; angela.leo@student.unisi.it (A.L.); erica.pranzini@unifi.it (E.P.); laura.pietrovito@unifi.it (L.P.); elisa.pardella@student.unisi.it (E.P.); matteo.parri@unifi.it (M.P.); paolo.cirri@unifi.it (P.C.); silvia.peppicelli@unifi.it (S.P.); eugenio.torre@unifi.it (E.T.); paola.chiarugi@unifi.it (P.C.); giovanni.raugei@unifi.it (G.R.); 2 Division of Pediatric Oncology/Hematology, Meyer University Children’s Hospital, 50139 Florence, Italy; gennaro.bruno@unifi.it (G.B.); maura.calvani@meyer.it (M.C.); 3 Department of Dermatology, Emory School of Medicine, Winship Cancer Institute, Atlanta, GA 30322, USA; mpapke@emory.edu (M.S.); lyang02@emory.edu (L.Y.); lei.zhu@emory.edu (L.Z.); jarbise@emory.edu (J.L.A.); 4 Atlanta Veterans Administration Medical Center, Decatur, GA 30033, USA; 5 Department of Experimental and Clinical Medicine, University of Florence, Viale Morgagni 50, 50134 Florence, Italy

**Keywords:** melanoma, amoeboid motility, Claisened Hexafluoro, mitochondria

## Abstract

**Simple Summary:**

Metastatic melanoma is one of the most aggressive and lethal malignancies with a poor prognosis. Several data underline the crucial role of amoeboid motility in the dissemination of metastatic melanoma cells. Thus, targeting this phenomenon could represent a promising strategy to prevent metastasis formation and improve metastatic melanoma patient’s survival. With this aim, we investigated the effect of Claisened Hexafluoro, a chemical analogue of Honokiol, on human metastatic melanoma cells. We demonstrated that Claisened Hexafluoro, by deregulating the mitochondrial activity, inhibits amoeboid motility and impairs many steps of the dissemination process, finally decreasing the in vivo metastatic spreading. Collectively, these data suggest possible future applications of Claisened Hexafluoro for the treatment of metastatic melanoma.

**Abstract:**

Metastatic melanoma is characterized by poor prognosis and a low free-survival rate. Thanks to their high plasticity, melanoma cells are able to migrate exploiting different cell motility strategies, such as the rounded/amoeboid-type motility and the elongated/mesenchymal-type motility. In particular, the amoeboid motility strongly contributes to the dissemination of highly invasive melanoma cells and no treatment targeting this process is currently available for clinical application. Here, we tested Claisened Hexafluoro as a novel inhibitor of the amoeboid motility. Reported data demonstrate that Claisened Hexafluoro specifically inhibits melanoma cells moving through amoeboid motility by deregulating mitochondrial activity and activating the AMPK signaling. Moreover, Claisened Hexafluoro is able to interfere with the adhesion abilities and the stemness features of melanoma cells, thus decreasing the in vivo metastatic process. This evidence may contribute to pave the way for future possible therapeutic applications of Claisened Hexafluoro to counteract metastatic melanoma dissemination.

## 1. Introduction

Cell motility is a physio-pathological process occurring during embryo-morphogenesis, wound repair, angiogenesis, and immune responses, as well as during different steps of the metastatic process [1]. Migration strategies can be divided into two main groups: single cell and collective motility. Among the first group, amoeboid and mesenchymal motilities are the most described and are strictly implicated in the metastatic process, even if endowed with different cell morphology, adhesive [2,3] and contractile abilities [4,5], Rho-family GTPase signaling activity [6,7], and cytoskeletal organization [8,9,10]. In particular, amoeboid motility provides cancer cells with a fast migratory phenotype, often associated with stem-like features [11]. Cells employing amoeboid motility are characterized by rounded morphology and are able to move rapidly through the fibers of the extracellular matrix (ECM) without the need for proteolytic enzymes production [2,12]. Moreover, amoeboid cells display a lack of polarization and a strong ability to contract the actin filaments which form cortical rings [8,13,14]. Key players in this process are the small GTPase RhoA and its downstream effectors, Rho-associated protein kinase (ROCK) and myosin light chain (MLC) [15,16].

Metastatic melanoma is one of the most aggressive and lethal malignancies with a poor prognosis. Until cancer cells remain localized in the site of primary tumor formation, melanoma patients generally experience a positive outcome. However, once melanoma cells spread to distant organs, mostly to the brain, lungs, liver, and small bowel, patients’ life expectancy strongly decreases. Even if in recent years new therapeutic approaches are increasing the overall survival of melanoma patients [17], new therapies aimed at targeting invasive cells and preventing their metastatic dissemination are urgently needed.

Targeting ameboid motility in melanoma could be a promising strategy to prevent metastatic dissemination. Indeed, melanoma cells are able to shift between mesenchymal and amoeboid motility, thus displaying remarkable migratory plasticity [18]. Amoeboid motility is more efficient and less energy-consuming than the mesenchymal strategy, thus potentiating cancer cell ability to migrate in the complex and dynamic ECM environment, even under stressful conditions, such as hypoxia [19]. For these reasons, dampening the amoeboid motility of melanoma cells may reduce cancer cells’ possibility to metastasize to distant organs, thereby strongly increasing the recovery of melanoma patients.

Claisened Hexafluoro (CH) is a chemical analogue of Honokiol (HNK), a biphenolic compound derived from Magnolia officinalis. For centuries, HNK has been used in traditional Chinese medicine for the treatment of a wide range of disorders, thanks to its biological effects, such as anti-inflammatory [20], anti-arrhythmic [21], anti-oxidative [22], neuroprotective [20,23], anti-angiogenic [24,25], anti-microbial [26,27,28], and anti-depressant [29,30] activities. Moreover, several studies have demonstrated the antitumoral efficacy of HNK in numerous cancer types, including hepatocellular carcinoma, lung, breast, head and neck, colon, and prostate cancers, and melanoma, both in vitro and in vivo [31,32]. Interestingly, HNK is also able to prevent the metastatic dissemination in breast cancer and melanoma [33,34].

Less is known about the antitumoral properties of CH, for which, to date, no evidence is available about the molecular targets and the mechanism of action. However, it has been recently demonstrated that CH can regulate the expression of SIRT3, mitigating organ fibrosis [35]. Furthermore, it can impair the in vivo proliferation of Vemurafenib-resistant melanoma cells by promoting an increase in reactive oxygen species (ROS) generation [36].

Starting from this evidence, we wondered whether CH could also act as a useful agent to treat metastatic melanoma. Here, we tested CH as an inhibitor of the amoeboid motility in vitro and tumor dissemination of melanoma cells in vivo. Our results may pave the way for future applications of this compound to fight melanoma cell invasion, thanks to its extraordinary effectiveness in blocking amoeboid motility.

## 2. Materials and Methods

### 2.1. Cell Lines and Materials

All the reagents were purchased from Sigma Aldrich (St. Louis, MO, USA), unless otherwise specified.

A375M6 and WM1361 melanoma cells were kindly donated by Lido Calorini from the Department of Experimental and Clinical Biomedical Sciences “Mario Serio”, University of Florence. A375M6 cells were isolated in Calorini’s laboratory from lung metastasis of SCID bg/bg mice, as previously described [37]. HS294T mesenchymal melanoma cells and HUVEC cells were purchased from ATCC. Cells were routinely grown in Dulbecco’s Modified Eagle’s Medium (DMEM) supplemented with 10% fetal bovine serum (FBS, Euroclone), 2 mM glutamine, 100 U/mL penicillin, and 100 µg/mL streptomycin, in a humidified atmosphere with 5% CO_2_ at 37 °C. WM1361 cells were cultured in MCDB 131 Medium/Leibovitz’s L-15 Medium (4:1) (Gibco, Thermofisher Scientific, Carlsbad, CA, USA). supplemented with 2% FBS, 5 μg/mL Insulin, 15 μg/mL Bovine Pituitary Extract (BPE), 1,68 mM CaCl_2_, 5 ng/mL EGF, 100 U/mL penicillin, and 100 µg/mL streptomycin. For A375M6, HS294T and WM1361 cells, the starvation medium consisted in their specific culture medium with no FBS supplementation. CH was provided by Jack L. Arbiser from Emory University. For all the experiments (unless otherwise specified), A375M6 and HS294T cells were serum-starved for 24 h, treated for additional 24 h with 10 μM CH and then analyzed. Besides, WM1361 cells were incubated for 24 h with 5 μM CH in starvation medium, before analysis.

Anti-RhoA (sc-418) and anti-β-Actin (sc-47778) antibodies were purchased from Santa Cruz Biotechnology (Dallas, TX, USA); anti-AMPK (#2532), anti-p-AMPK (Thr172) (#2535) antibodies were from Cell Signalling (Danvers, MA, USA); anti-EphA2 antibody was from Thermofisher Scientific (Carlsbad, CA, USA). Secondary antibodies were from Santa Cruz Biotechnology. Matrigel TM Basement Membrane Matrix (356234) was from BD Biosciences (San Jose, CA, USA). Recombinant human TNF-α (300-01A) was from Peprotech Inc (Rocky Hill, NJ, USA). CellTraceTM carboxyfluorescein succinimidyl ester (CFSE, C34554) was from Life Technologies. Rhotekin-RBD Human GST (RT01-A) was from Cytoskeleton, Inc. (Denver, CO, USA).

### 2.2. Cell Viability Assay

A375M6 cells (2 × 10^4^) and WM1361 cells (3 × 10^4^) were seeded in 24-multiwell plates and treated with or without increasing CH concentrations for 24, 48, and 72 h. Cell viability was evaluated by the addition of crystal violet solution (0.5% in 20% methanol). After 5 min of staining, the fixed cells were washed with phosphate-buffered saline (PBS) and solubilized with 200 µL/well of 0.1 M sodium citrate pH 4.2. The absorbance at 595 nm was evaluated using a microplate reader (BioRad, Hercules, CA, USA).

### 2.3. Western Blotting

Cells were lysed on ice in radioimmunoprecipitation assay (RIPA) buffer (50 mM TrisHCl pH 7.5, 150 mM NaCl, 100 mM NaF, 2 mM EGTA, 1% Triton X-100, 10 μL/mL protease and phosphatase inhibitors. 20 to 50 μg of total proteins were loaded on SDS-PAGE gels and transferred to PVDF membranes (Biorad, Hercules, CA, USA). Membranes were incubated overnight at 4 °C with the primary antibodies. After washing in PBS-Tween 20 (0.1%), membranes were incubated with the appropriate horseradish peroxidase-conjugated secondary antibodies for 1 h. Proteins were detected using Clarity Western ECL (BioRad, Hercules, CA, USA), and images were acquired using Amersham Imager 600 luminometer (Amersham, Buckinghamshire, UK). Quantification of bands was carried out by using the ImageJ software (NIH, USA).

### 2.4. RhoA Activity Assay

A375M6, WM1361, and HS2947 cells were directly lysed in RIPA buffer, the lysates were clarified by centrifugation, and RhoA-GTP levels were quantified. Briefly, lysates were incubated with 10 μg of Rhotekin–glutathione S-transferase (GST) absorbed on glutathione-Sepharose beads for 1 h at 4 °C. GST pulled-down immunoreactive RhoA was then quantified by Western blot analysis. Lysates were normalized for RhoA content by immunoblot.

### 2.5. Invasion Assay

A375M6, WM1361, or HS294T cells were treated or not with 10 μM CH for 24 h combined with or without 8 μM Compound C. Then, 5 × 10^5^ (A375M6 or WM1361) or 6 × 10^5^ cells (HS294T), were seeded onto Matrigel-precoated Boyden chamber (8-mm pore size, 6.5 mm diameter, 12.5 mg Matrigel/filter) in starvation medium. In the lower chamber, complete medium (FBS 10%) was used as chemoattractant. Following 5 h for A375M6 cells and 24 h for WM1361 and HS294T cells, the non-invading cells on the upper surface of the transwell were removed with a cotton swab. The filters were then stained using the Diff-Quik kit (BD Biosciences, Franklin Lakes, NJ, USA), and photographs of randomly chosen fields were taken. The quantification was performed using ImageJ software (NIH, USA). For EphA2 silencing, A375M6 cells were transiently transfected with SureSilencing shRNA Plasmid (SuperArray, Frederick, MD, USA) using Lipofectamine 2000 (Thermofisher scientific, Waltham, MA, USA), as previously reported [38]. After 48 h, a Boyden chamber invasion assay was performed as mentioned above.

### 2.6. 3D Invasion Assay

We seeded 2 × 10^4^ A375M6 cells in 96-multiwell plates coated with 1.5% Agar. After 4 days, spheroids were obtained and photographed. Subsequently, Matrigel (50 μg/cm^2^) was added to each well, and the cells were treated or not with 10 μM CH. The photos were taken 24 h after the treatment. The invasive capacity was calculated by subtracting the inner area, which represents the initial spheroid, from the total area. The area was calculated with ImageJ software (NIH, Bethesda, MD, USA).

### 2.7. Cell Migration in Three-Dimensional Collagen Matrices

Sub confluent A375M6 or HS294T cells, treated or not with 10 μM CH for 24 h, were labeled with CFSE (360 ng/mL) and then detached by EDTA (2 mM), washed, incorporated into three-dimensional collagen lattice (1.67 mg/mL, collagen I, rat tail, BD biosciences, 354236) and monitored by time-lapse video microscopy [39]. For three-dimensional time-lapse confocal microscopy (Leica-SP5 system, Wetzlar, Germany), cells were scanned for 12 h at 3 min time intervals for simultaneous fluorescence and backscatter signal (reflection), and reconstructed. Three-dimensional motility of cells is shown by time lapse of xyzt analysis (three-dimensional analysis over time).

### 2.8. Adhesion Assay

We seeded 1 × 10^5^ A375M6 and 5 × 10^4^ WM1361 cells in 24-multiwell plates coated with 300 μg/mL collagen (collagen I, rat tail, BD biosciences, Franklin Lakes, NJ, USA, 354236) or 2.5 μg/cm^2^ fibronectin (F2006) for 10 min. Adherent cells were fixed with methanol, and photos of the wells were taken. Cells were counted using the ImageJ software (NIH, USA). Adhesion to endothelium assay was performed as previously described [40]. Briefly, A375M6 cells, stained with CFSE (360 ng/mL), were seeded onto a transwell precoated with 5 × 10^4^ HUVECs, previously activated with 10 ng/mL TNFα for 90 min, and allowed to adhere for 30 min.

### 2.9. Trans-Endothelial Migration Assay

HUVECs were seeded the day before the experiment, grown to confluence on the separating filter of a Boyden chamber (8 mm pore size, 6.5 mm diameter) and were activated with 10 ng/mL TNFα for 90 min. Thereafter, culture media were changed with fresh media and cells incubated for additional 2.5 h. A375M6 cells, stained with CFSE (360 ng/mL), were seeded onto HUVECs monolayer and allowed to migrate toward complete medium (FBS 10%) for 16 h. The non-invading cells were removed on the upper surface with a cotton swab and the cells fixed with methanol 70%. The number of migrated cells to the filter’s lower side was evaluated by counting fluorescent cells using an inverted fluorescent microscope and quantified with Image J software (NIH, USA).

### 2.10. Flow Cytometric Analysis

A375M6 cells were serum-starved for 24 h and treated with 10 μM CH for 48 h. Cells were then detached with Accutase and stained with anti-CD133-APC (566,596, BD Biosciences, Franklin Lakes, NJ, USA) antibody (CD133 antibody excitation/emission 496/576) for 30 min and analyzed through flow cytometry analysis. For mitochondrial superoxide detection, A375M6 and WM1361 cells were incubated for 10 min at 37 °C with 5 μM MitoSox (Thermofisher scientific, Waltham, MA, USA) in PBS. Cells were detached with Accutase, centrifuged, washed with PBS, and resuspended in 300 μL PBS. A flow cytometer analysis was then performed (MitoSox excitation/emission: 510/580 nm). For mitochondrial analysis, 30 nM MitoTracker Green (M36008, Thermofisher Scientific, Waltham, MA, USA) (Mito Tracker Green excitation/emission 490/516 nm) and 200 nM Tetramethylrhodamine Ethyl Ester, Perchlorate (TMRE, Thermofisher scientific, Waltham, MA, USA, T669) (TMRE excitation/emission 552/574 nm) were used according to manufacturers’ instructions. Cells were analyzed by flow cytometry using FACScan flow cytometer (BD Biosciences, Franklin Lakes, NJ, USA).

### 2.11. Confocal Image Acquisition and Analysis

A375M6 cells were stained with MitoTracker Green (M36008, Thermofisher Scientific, Waltham, MA, USA,) probe and samples were examined using a confocal microscope (TCS SP5; Leica). Fluorescence quantification and 3D reconstruction were assessed using ImageJ (NIH, USA) and Imaris Bitplane software (Belfast, UK), respectively.

### 2.12. RT-PCR

Total RNA was purified from A375M6 cells using the RNeasy mini Kit (Qiagen, Hilden, Germany) according to the manufacturer’s instructions. The reverse transcription reaction was performed with QuantiTect Reverse Transcription Kit (Qiagen, Hilden, Germany) using 1 μg of total RNA. mRNA expression was performed using QuantiFast SYBR Green (Qiagen, Hilden, Germany). The primers used were: NANOG: 5′-ACCTTGGCTGCCGTCTCTGG-3′ (forward), 5′-AGCAAAGCCTCCCAATCCCAAACA-3′ (reverse); KLF4: 5′-GCAGCCACCTGGCGAGTC TG-3′ (forward), 5′-CCGCCAGCGGTTATTCGGGG-3′ (reverse); OCT3/4: 5′-TTTTGGTACCCCAGGCTATG-3′ (forward), 5′-GCAGGCACCTCAGTTTGAAT-3′ (reverse) and CD271: 5′-CGAGGCACCACCGACAACCT-3′ (forward), 5′-TGGTTCACTGGCCGGCTGTT-3′ (reverse). β2 was used as normalizer. All the amplifications were run on 7500 Fast Real-Time PCR System (BioRad, Hercules, CA, USA). Data were reported as relative quantity with respect to the calibrator sample using the −ΔΔ 2 Ct method.

### 2.13. Melanosphere Formation

A375M6 cells were detached using Accutase and plated at 1000 cells/plate on low attachment 100 mm plate in serum-free DMEM/F12 1:1 (Thermo Fisher Scientific, Waltham, MA, USA) supplemented with N2 (Thermo Fisher Scientific, Waltham, MA, USA), 0.6% glucose, 20 μg/mL insulin (Eli Lilly Indianapolis, IN, USA), 10 ng/mL b-FGF, and 10 ng/mL EGF. Cells were grown under these conditions for 15 days to obtain melanospheres. For the evaluation of self-renewal, a single melanosphere was dissociated in single cells with Accutase and a dilution of one cell per well into 96-well low attachment plates was performed without CH treatment in order to isolate individual P1 melanospheres.

### 2.14. Seahorse XFe96 Metabolic Assays

A375M6 and WM1361 cells, seeded in XFe96 cell culture plates with 3 × 10^5^ cells per well, were treated or not with 10 μM CH for 24 h. The medium was then replaced with XF base medium (Agilent, Santa Clara, CA, USA) supplemented with 25 mM glucose, 2 mM glutamine, and 1 mM sodium pyruvate. Cells were then incubated for 1 h at 37 °C in a non-CO_2_ incubator before the analysis and XF Mito Stress Test (Agilent, Santa Clara, CA, USA) was performed. The analysis revealed basal respiration, maximal respiration, and the cells’ ability to exploit mitochondrial oxidative metabolism. This analysis was performed by real-time measurement of extracellular acidification (ECAR) and of oxygen consumed (OCR) after the injection of a sequence of compounds that interfere with the electron transport chain: oligomycin (1 µM), carbonyl cyanide-4 (trifluoromethoxy) phenylhydrazone (FCCP) (1 µM), and Rotenone/Antimycin A (0.5 µM). Protein quantification was used to normalize the results.

### 2.15. Lactate Quantification Assay

A375M6 cells were treated for 24 h with or without 10 μM CH and the Lactate Colorimetric Assay Kit (Biovision, Milpitas, CA, USA) was performed according to the manufacturer’s instructions. All data were normalized on cell protein content.

### 2.16. AMP/ATP Quantification Assay

AMP/ATP Ratio Colorimetric Assay Kit (Biovision, Milpitas, CA, USA) was utilized according to the manufacturer’s instructions on A375M6 cells. All data were normalized on cell protein content.

### 2.17. Lung Colonization Assay

The tail vein injection method was developed and approved by the Institutional Animal Care and Use Committee (IACUC) of Emory University (PROTO201800258 approved on 2/14/2019), and all in vivo experiments were performed in accordance with the approved IACUC protocol guidelines and regulations. Male NU-Foxn1nu mice (8 weeks old, 5 animals per group) were injected with 1 × 10^6^ A375M6 cells into the tail veins. The mice were treated for 8 weeks with or without CH: 3 mg/animal/day, 5 times a week in 30% Intralipid. CH was dissolved in 100% ethanol at 100 mg/mL, diluted 1:10 in 30% intralipid, and 300 μL was injected intraperitoneally (IP). CH was prepared fresh. Lungs were paraffin embedded, sectioned in slices of 4 µm thickness and stained with hematoxylin and eosin (H&E). Ten slices from each lung were examined. Lungs were inspected for metastatic nodules by histological analysis after H&E and Masson-Fontana (Bio-Optica, Milano, Italy) staining. Histological images were acquired using a Nikon Eclipse microscope.

### 2.18. Statistical Analysis

Statistical analysis of the data was performed by unpaired Student t-test for pairwise comparison of groups and with one-way ANOVA for multiple comparison of groups if not specified. All data were expressed as the mean ± SEM. A *p* value ≤ 0.05 was considered statistically significant. All the statistical analyses were carried out on three biological replicates.

## 3. Results

### 3.1. CH Inhibits Amoeboid Motility and Invasive Ability of Melanoma Cells

**Figure 1:**
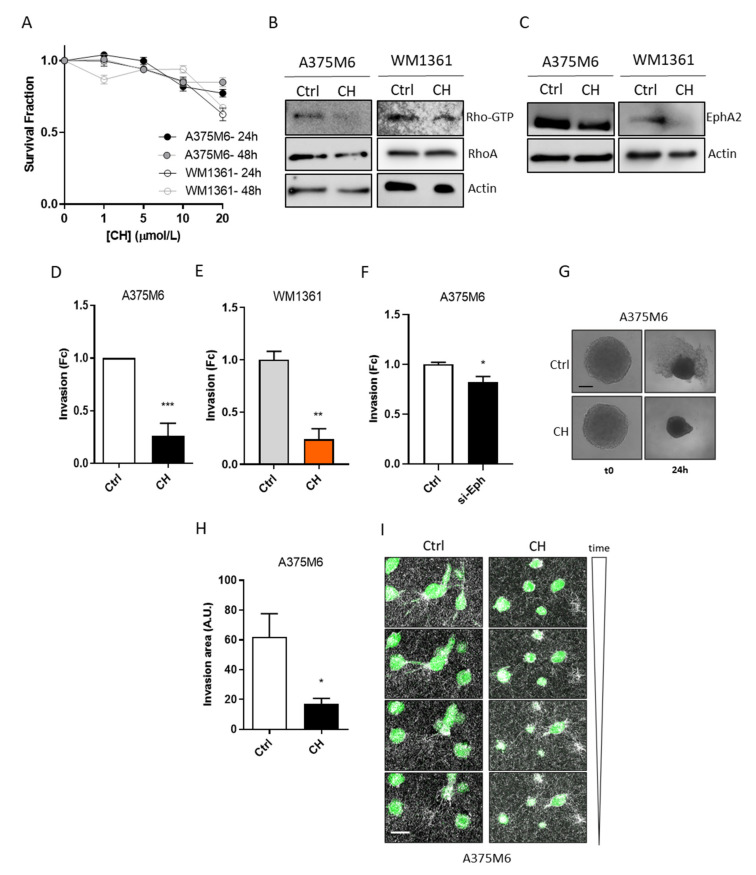
Treatment with CH decreases amoeboid motility of A375M6 and WM1361 melanoma cells. (**A**) Cell viability of amoeboid melanoma cells under CH treatment. A375M6 and WM1361 cells were treated for 24 h and 48 h with increasing CH concentrations and stained with crystal violet to determine the maximum non-toxic dose of the drug. Data are reported as mean ± SEM from three independent experiments; one-way-ANOVA. (**B**) Impact of CH administration on RhoA activation. Pull-down assays were performed to detect the GTP-bound conformation of RhoA in A375M6 and WM1361 melanoma cells, treated for 24 h with 10 µM or 5 µM CH, respectively. Anti-total RhoA and anti-actin immunoblots were performed to assess equal protein loading. Images are representative of three biological replicates. (**C**) EphA2 protein levels in amoeboid melanoma cell lines following CH treatment. Protein lysates from A375M6 and WM1361 melanoma cells, treated for 24 h with 10 µM or 5 µM CH, respectively, were analyzed by western blotting using the anti-EphA2 antibody. Anti-actin immunoblot was performed to assess equal protein loading. Images are representative of three biological replicates. (**D**,**E**) Invasion abilities of A375M6 and WM1361 cells assessed by Boyden Chamber invasion assay. A375M6 and WM1361 cells were treated for 24 h with 10 µM or 5 µM CH, respectively. Then, 5 × 10^4^ cells were seeded in the upper compartment of a Boyden chamber coated with Matrigel and allowed to invade toward complete medium (FBS 10%). Cell invasion was evaluated after Diff-Quick staining by counting cells in three randomly chosen fields. Data are reported as mean ± SEM from three independent experiments; *t*-test; ** *p* < 0.01, *** *p <* 0.001. (**F**) Impact of EphA2 on the invasive potential of A375M6 cells. EphA2 silenced A375M6 cells were seeded in the upper compartment of a Boyden chamber coated with Matrigel and allowed to invade for 5 h toward complete medium (FBS 10%). Cell invasion was evaluated after Diff-Quick staining by counting cells in three randomly chosen fields. Data are reported as mean ± SEM from three independent experiments; *t*-test; * *p <* 0.05. (**G**,**H**) 3D invasion assay was performed by seeding A375M6 cells in 96-well plate and, following the spheroid formation (4 days), Matrigel was added in each well and cells were treated or not with 10 µM CH for 24 h. Then, pictures were taken, and invasion area was calculated by subtracting the inner spheroid area from the total area using Image J software. Images are representative of three biological replicates. Scale bar: 100 µM. Data are reported as mean ± SEM from three independent experiments; *t*-test; * *p <* 0.05. (**I**) Migration of A375M6 melanoma cells in three-dimensional collagen lattice. Cells were treated with 10 μM CH for 24 h. CFSE labeled cells were incorporated into three-dimensional collagen I lattices and monitored by confocal fluorescence-reflection video microscopy for 12 h. Melanoma cells are reported in green, whereas the back scatter signal of the collagen I is reported in white. Images are representative of three independent experiments. Scale bar: 10 µM.

To test CH as a potential inhibitor of amoeboid motility, we utilized the human highly metastatic melanoma A375M6 cell line and the amoeboid melanoma WM1361 cell line [6,41]. Highly invasive metastatic melanoma cells are characterized by increased amoeboid motility [42,43,44,45]. First, we performed a crystal violet assay to determine the maximal concentration of CH displaying non-toxic effects on A375M6 and WM1361 cells after 24 h and 48 h of treatment (Figure 1A). On the basis of the obtained results, we identified CH 10 µM for A375M6 and 5 µM for WM1361 to perform all the following experiments. Next, we tested the effect of CH treatment on two of the main amoeboid markers: the small GTPase RhoA activity and the expression of EphA2 (an upstream activator of RhoA) [18,46]. CH treatment induces a decrease of Rho-GTP (Figure 1B) and EphA2 protein expression levels (Figure 1C) in both cell lines. Conversely, no changes in EMT markers were observed following CH treatment indicating that the compound specifically targets amoeboid motility determinants (data not shown). In order to verify whether RhoA reduced activation correlates with a decrease in cell motility, we performed invasion assays with both Matrigel-coated Boyden chambers (Figure 1D,E) and 3D-cultures (Figure 1G,H). A375M6 and WM1361 cells, treated for 24 h with CH and then seeded in Matrigel-coated Boyden chambers, were let to migrate towards complete medium in the absence of the treatment. As expected, cells show a substantial decrease in their invasion abilities following CH administration (Figure 1D,E). Accordingly, EphA2 silencing in A375M6 cells leads to a decrease in cell invasion, suggesting that CH could target the RhoA/ROCK axis (Figure 1F). Interestingly, by testing the effect of CH treatment on the invasive abilities of the mesenchymal-like HS294T cells [12], we did not observe significant effects on either Rho-GTP (Supplementary Figure S1A) or EphA2 protein expression levels (Supplementary Figure S1B), as well as in the invasion potential (Supplementary Figure S1C). These results indicate that CH specifically blocks amoeboid motility without affecting mesenchymal moving cells. Besides, the 3D invasion assay performed on spheroids grown on agar-Matrigel support confirmed a substantial decrease in the A375M6 cell invasion abilities following CH treatment (Figure 1G,H).

To corroborate the obtained results, we monitored the motility of A375M6 cells in a 3D collagen I matrix by time-lapse confocal video microscopy. Cells, treated with or without CH for 24 h, were seeded in a collagen matrix and let to invade for 12 h. Images clearly show that CH-treated cells almost completely lose their ability to move with amoeboid motility and squeeze between the collagen fibers (Figure 1I and Video S1). By contrast, HS294T cells are able to move with a mesenchymal-type motility by degrading collagen fibers, both in the presence and absence of CH treatment (Supplementary Figure S1D and Video S2). Together, these results indicate that CH treatment effectively reduces cell motility by acting as a specific inhibitor of the amoeboid phenotype of metastatic melanoma cells.

### 3.2. CH Decreases Adhesion Abilities and Trans-Endothelium Migration of Amoeboid Melanoma Cells

**Figure 2:**
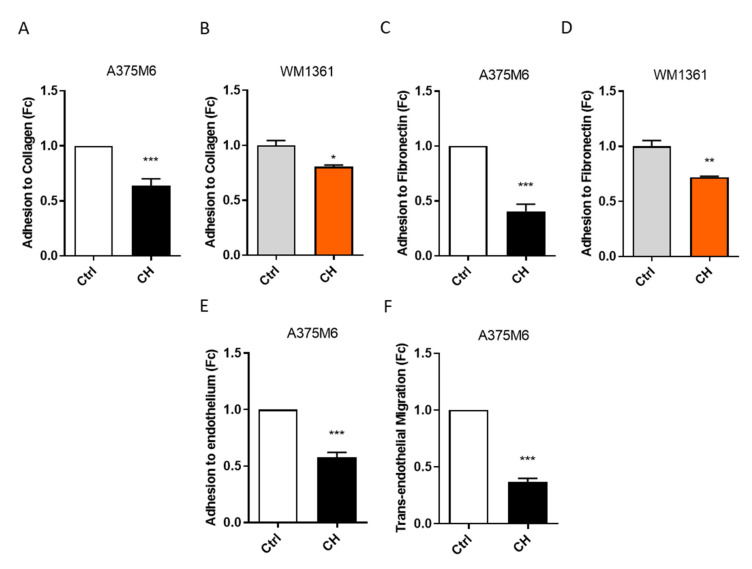
CH inhibits the adhesion to the ECM and trans-endothelium migration ability of amoeboid melanoma cells. (**A**,**B**) Amoeboid melanoma cell adhesion to collagen substrate. A375M6 (**A**) and WM1361 (**B**) cells, treated for 24 h with 10 µM or 5 µM CH, respectively, were let to adhere for 10 min to culture plates, previously coated with collagen. Adherent cells were stained with crystal violet and manually counted for quantification. Data are reported as mean ± SEM from three independent experiments; *t*-test; * *p <* 0.05, *** *p <* 0.001. (**C**,**D**) Amoeboid melanoma cell adhesion to fibronectin substrate. A375M6 (**C**) and WM1361 (**D**) cells, treated for 24 h with 10 µM or 5 µM CH, respectively, were let to adhere for 10 min to culture plates, previously coated with fibronectin. Adherent cells were stained with crystal violet and manually counted for quantification. Data are reported as mean ± SEM from three independent experiments; *t*-test; ** *p <* 0.01, *** *p <* 0.001. (**E**) Amoeboid melanoma cell adhesion to the endothelium. CFSE labeled A375M6 cells, treated or not with 10 μM CH for 24 h, were let to adhere for 30 min onto a monolayer of HUVEC cells. The adherent cells were visualized using an inverted fluorescent microscope and then quantified by counting CFSE-positive cells. Data are reported as mean ± SEM from three independent experiments; *t*-test; *** *p <* 0.001. (**F**) Amoeboid melanoma cell trans-endothelial migration ability. CFSE labeled A375M6 cells, treated or not with 10 μM CH for 24 h, were seeded onto a HUVEC monolayer in the upper compartment of a Boyden Chamber and let to migrate for 16 h towards complete medium (FBS 10%). Cell migration was evaluated by counting fluorescent cells in three randomly chosen fields acquired with inverted fluorescent microscope. Data are reported as mean ± SEM from three independent experiments, *t*-test; *** *p <* 0.001.

One of the earliest crucial steps in the metastatic dissemination process is the adhesion of cancer cells to the ECM [1]. Therefore, to test CH as a potential inhibitor of metastatic spreading, we performed adhesion assays using two main ECM components as supports; collagen and fibronectin. A375M6 and WM1361 cells were pre-treated for 24 h with CH and let to adhere for 10 min on culture plates previously coated with collagen or fibronectin. Results show that CH decreases the adhesion ability of A375M6 and WM1361 cells to both ECM components (Figure 2A–D, Supplementary Figure S2A,B).

The ability of tumor cells to enter the vessels and survive into the vasculature is a prerequisite for ensuring their metastatic spread. It has been demonstrated that amoeboid motility enables tumor cells to adhere to the endothelium and to increase their trans-endothelial migration capacity, thus further sustaining the metastatic process [40,47]. Therefore, to investigate if CH is able to inhibit this major metastatic step, we performed an endothelium adhesion assay and a trans-endothelial migration assay by seeding A375M6 cells onto a HUVEC’s monolayer and testing their ability to adhere and migrate through the monolayer. After CH treatment, we observed decreased endothelium adhesion ability (Figure 2E, Supplementary Figure S2C) and reduced trans-endothelial migration capacity (Figure 2F, Supplementary Figure S2D), suggesting that CH impairs melanoma cell potential to enter the blood vessels, spread to distant organs, and sustain the metastatic process.

### 3.3. CH Inhibits the Stemness Features of A375M6 Cells

The migratory plasticity of tumor cells has been correlated to the acquisition of stemness traits and the increase in metastatic abilities, which have been related for a long time to the epithelial-mesenchymal transition (EMT) of cancer cells [48]. However, the acquisition of the amoeboid phenotype has recently also been associated with increased stemness features in tumor cells [11,38]. This phenotype is often acquired by the most aggressive sub-populations within the tumor bulk [11,49]. To investigate whether CH is able to inhibit the stem-like phenotype of amoeboid melanoma cells, we analyzed the main melanoma stemness markers’ expression by FACS analysis and RT-PCR.

**Figure 3:**
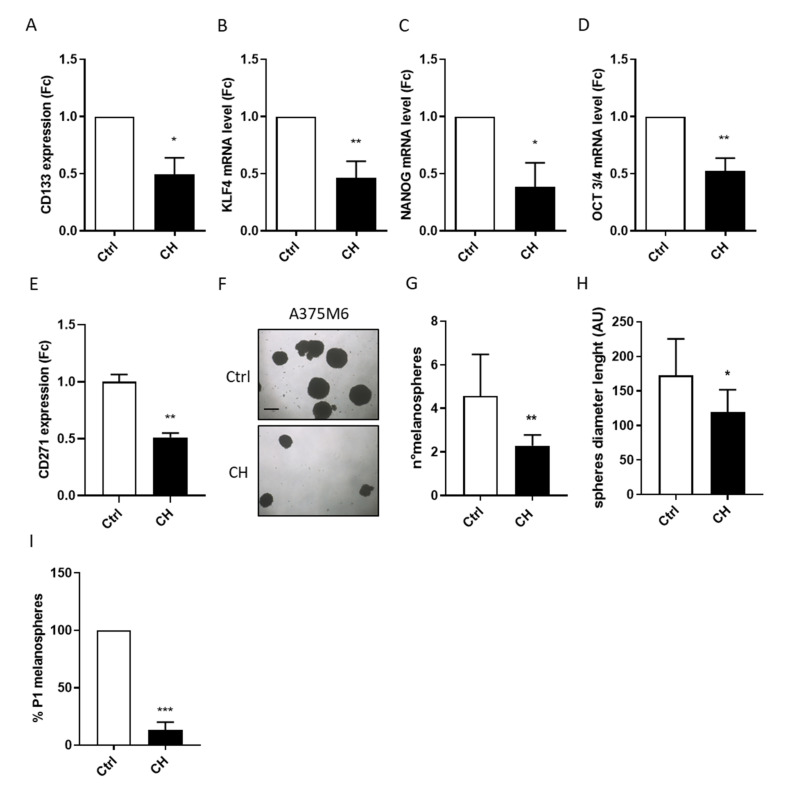
CH impairs the stem-like phenotype of A375M6 cells. (**A**) CD133 stemness surface marker expression levels in A375M6 melanoma cells after CH treatment. A375M6 cells were treated with 10 µM CH for 48 h and then CD133 expression levels were evaluated by FACS analysis. Data are reported as mean ± SEM from three independent experiments, *t*-test; * *p <* 0.05. (**B**–**E**) Major melanoma stemness marker expression levels in A375M6 cells after CH incubation. A375M6 cells were treated with 10 µM CH for 24 h and then KFL4 (**B**), NANOG (**C**), OCT3/4 (**D**), and CD27 (**E**) expression levels were analyzed by q-RT-PCR. Data are reported as mean ± SEM from three independent experiments, *t*-test; * *p* < 0.05, ** *p <* 0.01. (**F**,**G**) Melanosphere formation in A375M6 melanoma cells following CH treatment. A375M6 cells were treated with 10 μM CH for 24 h and then 1000 cells were seeded in a non-adherent culture plate. After 15 days, photos were taken and the number of melanospheres in the control and CH-treated samples was calculated as the mean of melanosphere number in eight randomly chosen fields for each sample. Representative images of three independent experiments are reported Scale bar: 200 µM. Data are reported as mean ± SEM from three independent experiments, *t*-test; ** *p <* 0.01. (**H**) Melanosphere dimensions. Melanospheres diameters were calculated in eight representative images of control and CH-treated samples. Data are reported as mean ± SEM, *t*-test; * *p <* 0.01. (**I**) P1 melanosphere formation in A375M6 cells after CH treatment. A375M6 cells were treated with 10 μM CH for 24 h and then 1000 cells were seeded in a non-adherent culture plate. After 15 days, primary melanospheres were collected, dissociated into single cell suspension, and re-seeded for P1 spheroid formation. After 10 days, P1 melanospheres were counted. Data are reported as mean ± SEM, *t*-test; *** *p <* 0.001.

We treated A375M6 cells with or without CH for 48 h, and we evaluated the stemness surface marker CD133 levels through FACS analysis. Results pointed out a decrease of about 40% in CD133 levels in treated cells when compared to the control ones (Figure 3A). Moreover, we analyzed the expression levels of KLF4, NANOG, OCT3/4, and CD271, which are genes involved in stemness features and are able to induce pluripotency in melanoma [50,51]. The obtained results show a decreased expression of all the selected markers in A375M6 cells following CH treatment (Figure 3B–E). Finally, we performed a melanosphere formation assay to confirm the stemness properties reduction after CH administration. This assay is based on stem cells’ ability to survive in non-adherent conditions and to self-renew in a serum-free culture medium [52]. The obtained results clearly indicate that the treatment with the compound decreases the number and the size of A375M6-derived melanospheres (Figure 3F–H). Moreover, the strong impairment in the formation of P1 individual spheres, derived from dissociated single melanospheres further demonstrates that CH affects the self-renewal potential of A375M6 cells (Figure 3I). Taken together, these findings suggest that CH acts as an inhibitor of the melanoma stem-like phenotype.

### 3.4. CH Inhibits the Mitochondrial Activity and ATP Production of Melanoma Cells

**Figure 4:**
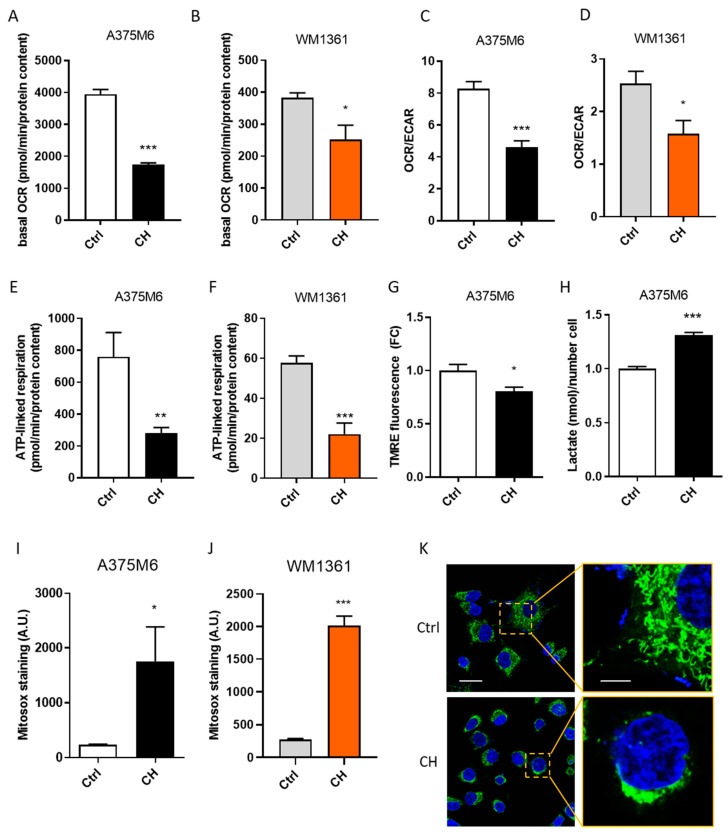
CH decreases the mitochondrial activity of A375M6 cells and increases mitochondrial ROS production. (**A**,**B**) Basal OCR, measured in real time with Seahorse XFe96 Mito Stress Test analysis on amoeboid melanoma cells after CH treatment. A375M6 (**A**) and WM1361 (**B**) cells were treated with 10 μM and 5 μM CH, respectively, for 24 h. Basal OCR was calculated as the OCR values before Oligomycin injection. Data are reported as mean ± SEM from four independent experiments, *t*-test; * *p* < 0.05, *** *p <* 0.001. (C, D) OCR/ECAR ratio measured in real time with Seahorse XFe96 Mito Stress Test analysis on amoeboid melanoma cells after CH treatment. A375M6 (**C**) and WM1361 (**D**) cells were treated with 10 μM CH for 24 h. The OCR/ECAR ratio was calculated by dividing basal OCR with the corresponding values of ECAR. Data are reported as mean ± SEM from four independent experiments, *t*-test; * *p* < 0.05, *** *p <* 0.001. (**E**,**F**) ATP-linked respiration measured in real time with Seahorse XFe96 Mito Stress Test analysis on amoeboid melanoma cells following CH treatment. A375M6 (**E**) and WM1361 (**F**) cells were treated with 10 μM CH for 24 h. The ATP-linked respiration was calculated by subtracting OCR values obtained following Oligomycin injection to basal OCR. Data are reported as mean ± SEM from four independent experiments, *t*-test; ** *p* < 0.01, *** *p <* 0.001. (**G**) Mitochondrial membrane potential of A375M6 cells following CH treatment. A375M6 cells were treated with 10 μM CH for 24 h. Mitochondrial membrane potential was analyzed by staining cells with TMRE probe, followed by FACS analysis. Data are reported as mean ± SEM from three independent experiments, *t*-test; * *p* < 0.05. (**H**) Extracellular lactate levels in A375M6 cells after CH treatment. Conditioned media of A375M6 cells, treated or not with 10 μM CH for 24 h, were collected and lactate concentration was measured with the Lactate Colorimetric Assay Kit. Data were normalized on cell number. Data are reported as mean ± SEM from three independent experiments, *t*-test; *** *p <* 0.001. (**I**,**J**) Mitochondrial ROS levels of amoeboid melanoma cells after CH treatment. Mitochondrial ROS levels were quantified by staining CH-treated and non-treated A375M6 (**I**) or WM1361 (**J**) cells with MitoSox probe and analyzed by FACS. Data are reported as mean ± SEM from three independent experiments, *t*-test; * *p* < 0.05, *** *p <* 0.001. (**K**) Mitochondrial morphology of A375M6 cells. A376M6, treated or not with 10 μM CH for 24 h, were stained with MitoTracker Green probe. Nuclei were labelled with Hoechst. Mitochondrial morphology was then analyzed by confocal microscopy. Images are representative of three biological replicates. Scale bar: 5 µm and 2 µm.

Several reports support the existence of a strong correlation between increased motility and altered mitochondrial metabolism in cancer cells [53]. Considering that HNK has been demonstrated to suppress mitochondrial respiration and increase the generation of ROS in the mitochondria [54], we asked whether similar effects may be driving CH-mediated inhibition of motility. Thus, we performed Seahorse XFe96 Mito Stress analysis on A375M6 and WM1361 cells pre-treated for 24 h with or without CH. Interestingly, the treatment induces a substantial decrease in the cellular oxygen consumption rate (OCR) both in basal conditions and after the treatment with different mitochondrial drugs (namely Oligomycin, FCCP, and Rotenone/antimycin A) (Figure 4A,B, Supplementary Figure S3A,B). Importantly, CH treatment does not affect extracellular acidification rate (ECAR) of A375M6 and WM1361 cells (Supplementary Figure S3C,D), indicating that it does not induce a global metabolic impairment, but a specific inhibition of the mitochondrial functionality. In keeping with these results, the OCR/ECAR ratio resulted to be decreased in CH treated cells (Figure 4C,D). In addition, the significant reduction in the ATP-linked OCR measured in treated cells further confirms that CH impairs mitochondrial functionality in both cell lines (Figure 4E,F). Moreover, treated cells also display a slighter increase in OCR following FCCP injection than non-treated cells, indicating a diminished spare respiratory capacity, defined as the difference between maximal and basal respiration (Supplementary Figure S3A,B). Measuring the mitochondrial membrane potential by staining cells with tetramethylrhodamine ethyl ester (TMRE), we found a slight decrease in the mitochondrial membrane potential of CH-treated A375M6 cells (Figure 4G), further confirming their impairment of the mitochondrial activity. In keeping with a decreased mitochondrial functionality, we expected that treated cells enhance glycolytic activity to overcome the lack of ATP availability. Indeed, we measured higher extracellular lactate release in A375M6 cells after 24 h of CH treatment (Figure 4H). Altogether, these data indicate that the treatment with CH affects mitochondrial functionality in metastatic melanoma cells.

Mitochondrial dysfunction frequently correlates with increased mitochondrial-ROS (mt-ROS) production [55]. To further confirm the CH-induced alteration in mitochondrial functionality, we assayed the mt-ROS content in treated and untreated cells. As expected, MitoSOX staining analysis (Figure 4I,J) pointed out a strong increase in the oxidative stress inside the mitochondria following CH treatment both in A375M6 and WM1361 cells. Dysfunctional mitochondria frequently display altered mass and structure [56]. Through MitoTracker Green staining we observed an increase in the mitochondrial mass measured by FACS analysis (Supplementary Figure S3E) and an alteration in the mitochondrial morphology (Figure, 4K) of A375M6 cells following CH treatment. Altogether, these data suggest that CH-treated cells undergo mitochondrial adaptations as a compensatory mechanism to overcome the mitochondrial dysfunction induced by the treatment.

### 3.5. CH Affects AMPK Signaling

A recent study described that HNK affects the activity of several protein kinases, including JNK, ERK, and p38 [57,58]. Interestingly, we observed similar effects on A375M6 melanoma cells after CH administration (Supplementary Figure S3F). Among them, AMPK emerged as one of the most interesting CH-regulated kinases. Indeed, alterations in mitochondrial energetics have been related to the AMPK signaling activation due to an impairment in the energy balance and a consequent increase in the AMP/ATP ratio [59]. AMPK is the main cellular energy status sensor and a key player in different cellular functions [60].

**Figure 5:**
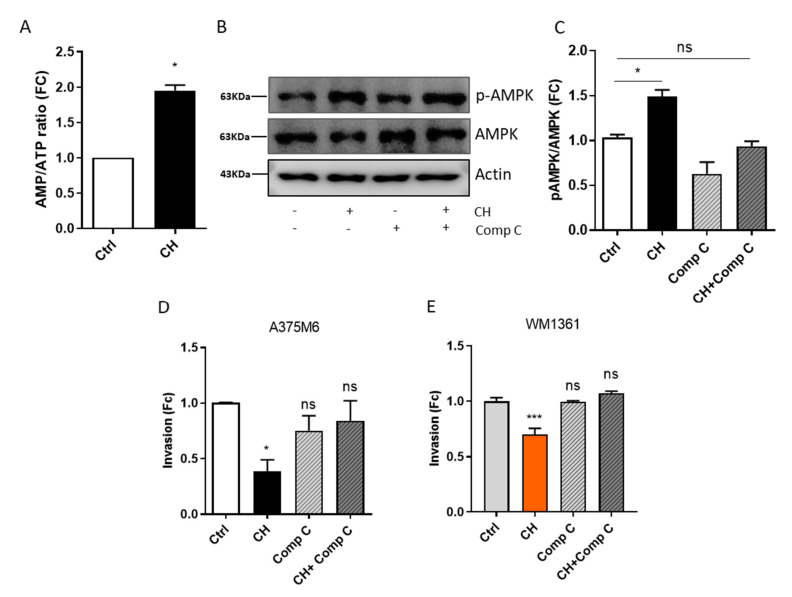
AMPK activity is involved in the inhibition of ameboid motility after treatment with CH. (**A**) AMP/ATP ratio in A375M6 cells after CH treatment. A375M6 cells were treated with 10 μM CH for 24 h and the AMP/ATP ratio was evaluated by AMP/ATP Ratio Colorimetric Assay Kit. Data are reported as mean ± SEM from three independent experiments, *t*-test; * *p* < 0.05. (**B**,**C**) AMPK activation status in A37M6 cells following CH treatment. Western Blot analysis was performed in A375M6 cells treated or not with 10 µM CH and/or 8 µM CC to test the phosphorylation levels of AMPK. Anti-total AMPK and anti-actin immunoblots were performed to ensure equal protein loading. Representative images of three independent experiments are reported. Data are reported as mean ± SEM from three independent experiments, one-way ANOVA; * *p* < 0.05. (**D**,**E**) Impact of AMPK inhibition by CH treatment on the invasive potential of amoeboid melanoma cells. A375M6 (**D**) and WM1361 (**E**) cells were treated or not with 10 µM or 5 µM CH and/or 8 µM CC, seeded in the upper compartment of a Boyden chamber coated with Matrigel and allowed to invade toward complete medium (FBS 10%). Cell invasion was evaluated after Diff-Quick staining by counting cells in three randomly chosen fields. Data are reported as mean ± SEM from three independent experiments; *t*-test; * *p <* 0.05, *** *p* < 0.001.

In order to evaluate whether CH-induced mitochondrial dysfunction also has an impact on the cellular energy status, we measured the AMP/ATP ratio in treated and untreated A375M6 cells. Interestingly, following CH treatment, it was possible to observe an increase in the AMP/ATP ratio (Figure 5A) indicating a condition of energy stress. Accordingly, in CH-treated A375M6 cells, we found increased AMPK phosphorylation (Thr-172) levels, indicating a rise in its activation (Figure 5B,C). Interestingly, AMPK pathway is implicated in several cellular processes, including cell motility, adhesion, and invasion [61]. To evaluate whether AMPK activation may mediate the reported decrease in cellular motility, we assayed cell invasion in the presence of the selective AMPK inhibitor, Compound C (CC) [62]. Interestingly, the CH-induced impairment of cell invasion is almost completely reversed in the presence of CC in A375M6 (Figure 5D) and WM1361 cells (Figure 5E), confirming the involvement of AMPK activation in CH-induced inhibition of cell motility.

### 3.6. CH Decreases the In Vivo Lung Metastasis Formation

**Figure 6:**
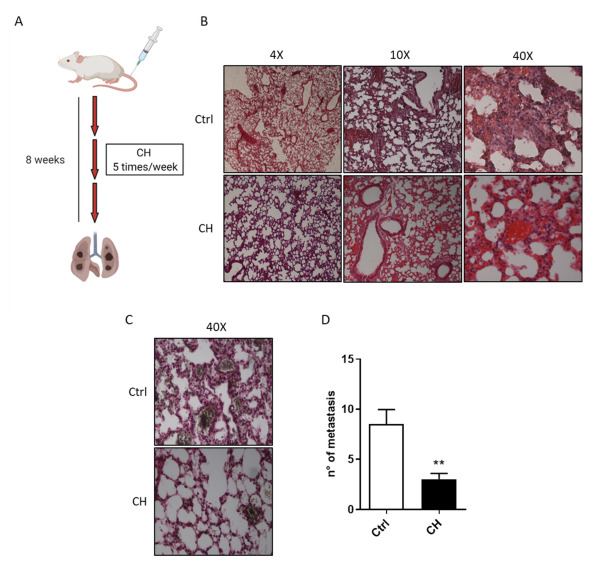
CH decreases lung colonization in NU-Foxn1numice Lung colonization assay. (**A**) Ten mice were injected into the lateral tail vein with 1 × 10^6^ A375M6 cells. Three mg/mouse/day in 30% intralipid CH was administered intraperitoneally 5 times a week to 5 mice. (**B**,**C**) Mice were sacrificed after 8 weeks and the lungs were inspected for lung health and metastatic nodules by histological analyses with H&E (**B**) and Masson Fontana staining (**C**). (**D**) Quantification of metastatic nodules in CH treated or not-treated mice. The mean number of metastatic nodules was counted in 10 slices per mice of paraffin-embedded tissue sections from lung metastases stained with H&E, *t*-test; ** *p <* 0.01.

Finally, to confirm whether CH affects A375M6 metastatic dissemination in vivo, we assayed its efficacy in an experimental model of metastasis using NU-Foxn1nu mice. A375M6 cells were injected into the tail vein of NU-Foxn1nu mice and CH was administrated to the treated group intraperitoneally 5 times a week for 8 weeks (Figure 6A). CH exposure does not alter the morphology of the lung parenchyma, as demonstrated by hematoxylin and eosin (H&E) staining of lung tissue sections (Figure 6B). Interestingly, H&E and Masson Fontana staining revealed that CH treatment results in a decrease in the total number of lung metastatic nodules (Figure 6B–D). These findings indicate that CH effectively inhibits lung colonization of circulating melanoma cells in vivo, without affecting the overall lung health, confirming the crucial role of CH-mediated inhibition of amoeboid motility in the in vivo metastatic process of melanoma cells.

## 4. Discussion

Cancer cell dissemination is one of the most harmful phenomena during cancer progression, frequently representing the leading cause of patients’ fatal outcome. Disseminating cancer cells modify their invasive abilities and metabolic properties [63] to adapt to changes occurring during the metastatic process, and to acquire enhanced resistance to both anoikis and anticancer therapies [64]. Altogether, these characteristics allow disseminating cells to overcome all the metastatic steps and facilitate the establishment of new metastatic lesions.

Metastatic melanoma accounts for the majority of skin cancer deaths. For this reason, halting melanoma dissemination through the development of new therapeutic approaches represents one of the major challenges in the clinic [65]. In this context, besides the successful therapies aimed at inhibiting oncogenes involved in melanoma and the efficacious immunotherapy, there is a real need for treatments that specifically target the metastatic process.

Different types of cell motility have been described in melanoma, such as the rounded/amoeboid-type motility, the elongated/mesenchymal-type motility, and the collective motility [6,14,66], supporting the idea that melanoma cells are highly plastic and can switch between different migration strategies, according to tumor microenvironmental conditions [6,66,67,68]. In particular, several studies have recognized the amoeboid motility as a detrimental feature of metastatic melanoma. Lorentzen and collaborators showed that a selected melanoma A375 population characterized by increased metastatic potential contains a higher proportion of cells (90%), which move through squeezing amoeboid motility than the less metastatic parental cell line [44]. Moreover, rounded cells predominate in the invasive front of melanoma, confirming the relevance of the amoeboid motility in driving the invasive process [42].

It is well known that the activation of Rho-ROCK signaling is required for rounded/amoeboid-type blebs-based movement, while it is not necessary for the elongated/mesenchymal-type protrusive one [6,66,69,70]. In keeping with these observations, several Rho GTPase proteins have been found overexpressed or mutated in metastatic tumors [71]. Furthermore, Misek and collaborators recently showed that the activation of RhoA family GTPases is present in ~50–60% of melanoma Vemurafenib-resistant cell lines in vitro, reinforcing the idea that this signaling pathway has a key role in the achievement of aggressive features by melanoma cells [72]. In this context, efforts have been made to arrest melanoma cell invasion by blocking Rho activity [45,73]. Considering the crucial role of amoeboid motility in driving melanoma metastasis, we investigated the possibility of using CH, a fluorinated synthetic HNK analogue, to treat highly metastatic melanoma. In this study, we demonstrated that CH is efficient in blocking the amoeboid motility of metastatic melanoma cells by decreasing EphA2 expression and RhoA activation. Accordingly, we observed a reduction in melanoma cell invasion ability in both 2D and 3D assays. Interestingly, CH does not induce a shift towards the mesenchymal style. Altogether, the aforementioned features suggest that CH displays a real therapeutic potential for metastatic melanoma treatment.

Moreover, our results show that CH can also inhibit several steps of the metastatic process, especially the trans-endothelial migration, a feature necessary to extra/intravasate during the dissemination process. Coherently, this phenomenon is mainly based on cancer cells’ ability to pass through the endothelial barriers with an amoeboid motility style. The inhibition of melanoma cells trans-migration is strictly correlated with the observed decrease in the number of metastatic nodules in vivo. Moreover, we demonstrate that CH inhibits both the stemness phenotype of melanoma cells and the ability to form melanospheres. Accordingly, previous results demonstrated that the achievement of amoeboid motility is characterized by increased stemness and clonogenic features in melanoma cells [11]. Alterations in cancer cell motility are frequently associated with metabolic reprogramming allowing cells to support the energy-consuming process of cell migration [63]. Due to mitochondria impairment, we observe a shift from OXPHOS to glycolysis, as revealed by increased lactate production, to sustain the energy demand of melanoma cells following CH treatment. Indeed, CH decreases OCR levels and mitochondria-related ATP production, induces mitochondrial depolarization, and increases mtROS accumulation. Of particular interest is the effect of CH on cellular redox status, as it has been demonstrated that increased mtROS levels are related to mitochondrial fusion and further ROS amplification, leading to the selective impairment of melanoma cell aggressiveness [74]. Thanks to its detrimental effects on mitochondrial functionality, CH could be of great utility in the treatment of tumors dependent on mitochondrial respiration, similarly to what is already observed for HNK [75]. Targeted therapy with MAPK-signaling inhibitors represents the leading chemotherapeutic approach for melanoma [76]. Interestingly, cells resistant to this type of therapy usually shift their metabolism from glycolysis to OXPHOS [77,78]. Thus, the concomitant treatment of CH with MAPK-signaling inhibitors could be beneficial in increasing the efficacy of these targeted therapies. Accordingly, Bonner and collaborators reported that CH effectively decreases proliferation of Vemurafenib resistant melanoma cells [36], which frequently display an OXPHOS-dependent metabolism [79,80].

Mechanistically, the reduction of the respiratory chain function following CH treatment is strictly associated with the increase in both AMP/ATP ratio and the consequent activation of the AMPK signaling. Our data reveal that the inhibition of cell invasion induced by CH is strictly dependent on AMPK phosphorylation/activation, as it almost completely reverted treating cells with the AMPK inhibitor CC [62]. Indeed, AMPK signaling, acting as the “sensor” of the cellular energy status, plays crucial roles in regulating cell migration. Indeed, Gayard and co-workers showed that AMPK is able to phosphorylate RhoA on Ser188 and to subsequently restrain the RhoA/ROCK signaling [81]. Accordingly, Guo and co-authors demonstrated that in melanoma cells AMPK-mediated inhibition of RhoA decreases cell invasion and migration [82]. In this light, the AMPK/RhoA/ROCK axis proved to be an indirect target of CH. Indeed, our data demonstrated that the mitochondrial dysfunction caused by CH treatment, sustains the AMPK signaling, finally leading to a decrease in RhoA activation and to an impairment of amoeboid motility.

In conclusion, although data herein presented have been obtained mainly in vitro and, up to date, only preliminary in vivo data are available, we believe that this study has a promising translational value. Indeed, CH may have potential clinical applications in the future.

Collectively, our data suggest that CH could be a powerful tool to counteract the amoeboid motility and the metastatic dissemination of melanoma cells by affecting the mitochondrial-mediated activation of AMPK and consequently blocking the RhoA/ROCK-dependent amoeboid phenotype.

## 5. Conclusions

This study shows the efficacy of CH in inhibiting amoeboid motility of metastatic melanoma cells, thus paving the way for a possible future clinical application of this compound to halt the metastatic disseminating process.

## Data Availability

The data presented in this study are available in the article and Supplementary Material.

## Supplementary Materials

The following are available online at https://www.mdpi.com/article/10.3390/cancers13143551/s1, Figure S1: Impact of CH treatment on HS294T mesenchymal-like melanoma cells, Figure S2: A375M6 melanoma cell adhesion, Figure S3: Mitochondrial functionality of A375M6 and WM1361 cells, Video S1: A375M6 cell migration in three-dimensional collagen matrices, Video S2: HS294T cell migration in three-dimensional collagen matrices.
