# Supplementary material for: Claisened Hexafluoro Inhibits Metastatic Spreading of Amoeboid Melanoma Cells
Source: Cancers (Basel). 2021 Jul 15;13(14):3551. doi: 10.3390/cancers13143551 (PMC8305480; doi:10.3390/cancers13143551)
Supplement: Supplementary file 1 [file cancers-13-03551-s001.zip › Cancers-1096282-supplementary/Supplementary_Figures/SuppFig.1.pdf]

Suppl. Fig.1

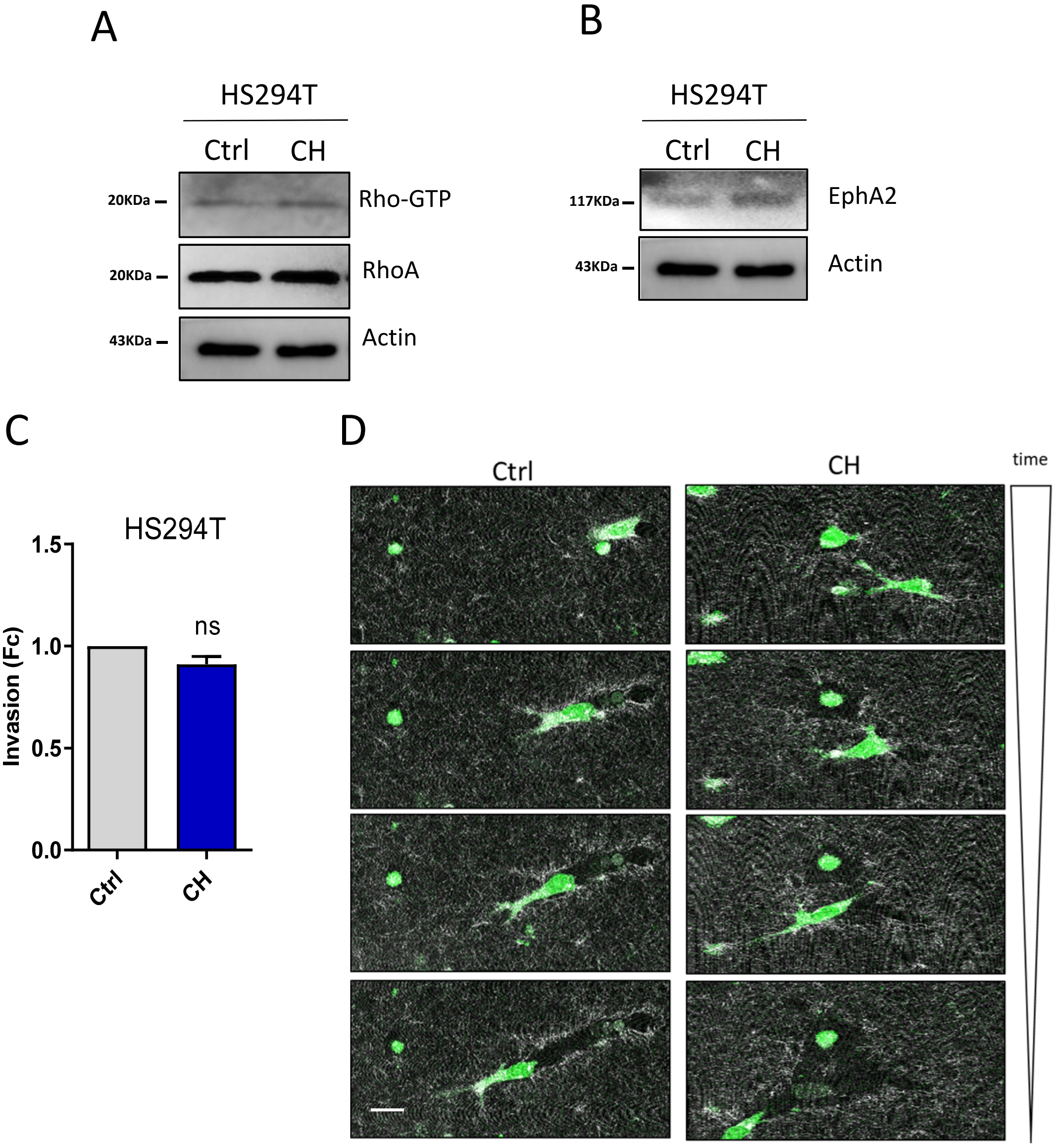

Supplementary Figure 1. (A) Impact of CH treatment on RhoA activation in HS294T mesenchymal-like melanoma cells. RhoA activation was evaluated through pull-down assay on HS294T cells, following treatment with 10 μM CH for 24 h. Anti-total RhoA and anti-actin immunoblots were performed to assess equal protein loading. Images are representative of three independent experiments. (B) EphA2 protein levels in mesenchymal-like HS294T melanoma cell lines following CH treatment. Protein lysates from HS294T cells, treated for 24 h with 10 μM CH, were analyzed by western blotting using the anti-EphA2 antibody. Anti-actin immunoblot was performed to assess equal loading. Images are representative of three independent experiments. (C) Invasion abilities of HS294T cells assessed by Boyden Chamber invasion assay. HS294T cells were treated for 24 h with 10 μM CH. Then, 5x104 cells were seeded in the upper compartment of a Boyden chamber coated with Matrigel and allowed to invade for 24 h toward complete medium (FBS 10%). Cell invasion was evaluated after Diff-Quick staining by counting cells in three randomly chosen fields. Data are reported as mean ± SEM from three independent experiments; t-test. (D) Migration of HS294T melanoma cells in three-dimensional collagen lattice. HS294T cells were treated with 10 μM CH for 24 h. CFSE labeled cells were incorporated into three-dimensional collagen I lattices and monitored by confocal fluorescence-reflection video microscopy for 16h. Melanoma cells are reported in green, whereas the back scatter signal of the collagen I is reported in white. Images are representative of three independent experiments. Scale bar: 10μM.
