# Supplementary material for: Claisened Hexafluoro Inhibits Metastatic Spreading of Amoeboid Melanoma Cells
Source: Cancers (Basel). 2021 Jul 15;13(14):3551. doi: 10.3390/cancers13143551 (PMC8305480; doi:10.3390/cancers13143551)
Supplement: Supplementary file 1 [file cancers-13-03551-s001.zip › Cancers-1096282-supplementary/Supplementary_Figures/SuppFig.2.pdf]

Suppl. Fig.2

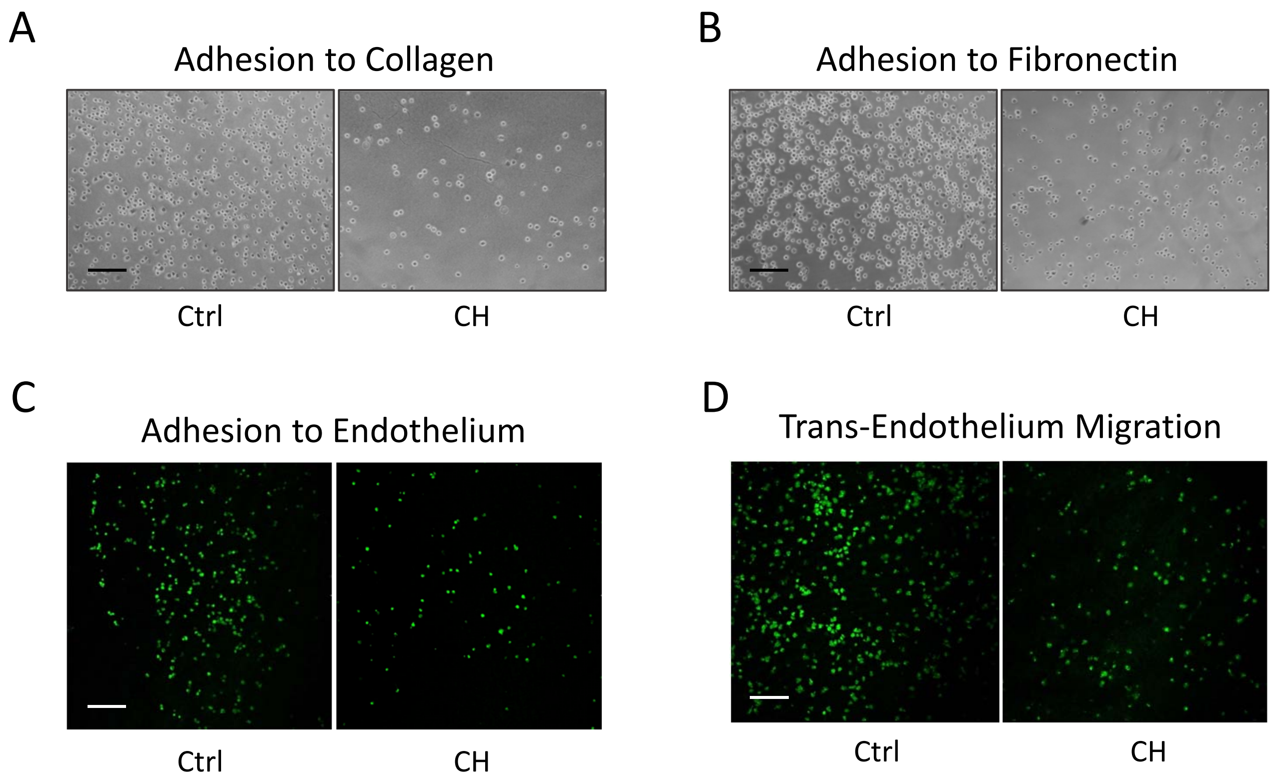

Supplementary Figure 2. (A) A375M6 melanoma cell adhesion to collagen substrate. A375M6 cells, treated for 24 h with 10 µM CH, were let to adhere to culture plates previously coated with collagen for 10 min. Images are representative of five randomly chosen fields. Scale bar: 100µM (B) A375M6 melanoma cell adhesion to fibronectin substrate. A375M6 cells, treated for 24 h with 10 µM CH, were let to adhere to culture plates previously coated with fibronectin for 10 min. Images are representative of five randomly chosen fields. Scale bar: 100µM. (C) A375M6 melanoma cell adhesion to the endothelium. CFSE labeled A375M6 cells, treated with 10 µM CH for 24 h, were let to adhere for 30 min onto a monolayer of HUVEC cells. The adherent cells were visualized using an inverted fluorescent microscope and representative images are reported. Scale bar: 100µM. (D) A375M6 melanoma cell trans-endothelial migration ability. CFSE labeled A375M6 cells, treated with 10 µM CH for 24 h, were seeded onto a HUVEC monolayer in the upper compartment of a Boyden Chamber and let to migrate for 16 h toward complete medium (FBS 10%). Photos of fluorescent cells of five randomly chosen fields were acquired with inverted fluorescent microscope and representative images are reported. Scale bar: 100µM .
