# Supplementary material for: Claisened Hexafluoro Inhibits Metastatic Spreading of Amoeboid Melanoma Cells
Source: Cancers (Basel). 2021 Jul 15;13(14):3551. doi: 10.3390/cancers13143551 (PMC8305480; doi:10.3390/cancers13143551)
Supplement: Supplementary file 1 [file cancers-13-03551-s001.zip › Cancers-1096282-supplementary/Supplementary_Figures/SuppFig.3.pdf]

Suppl. Fig.3

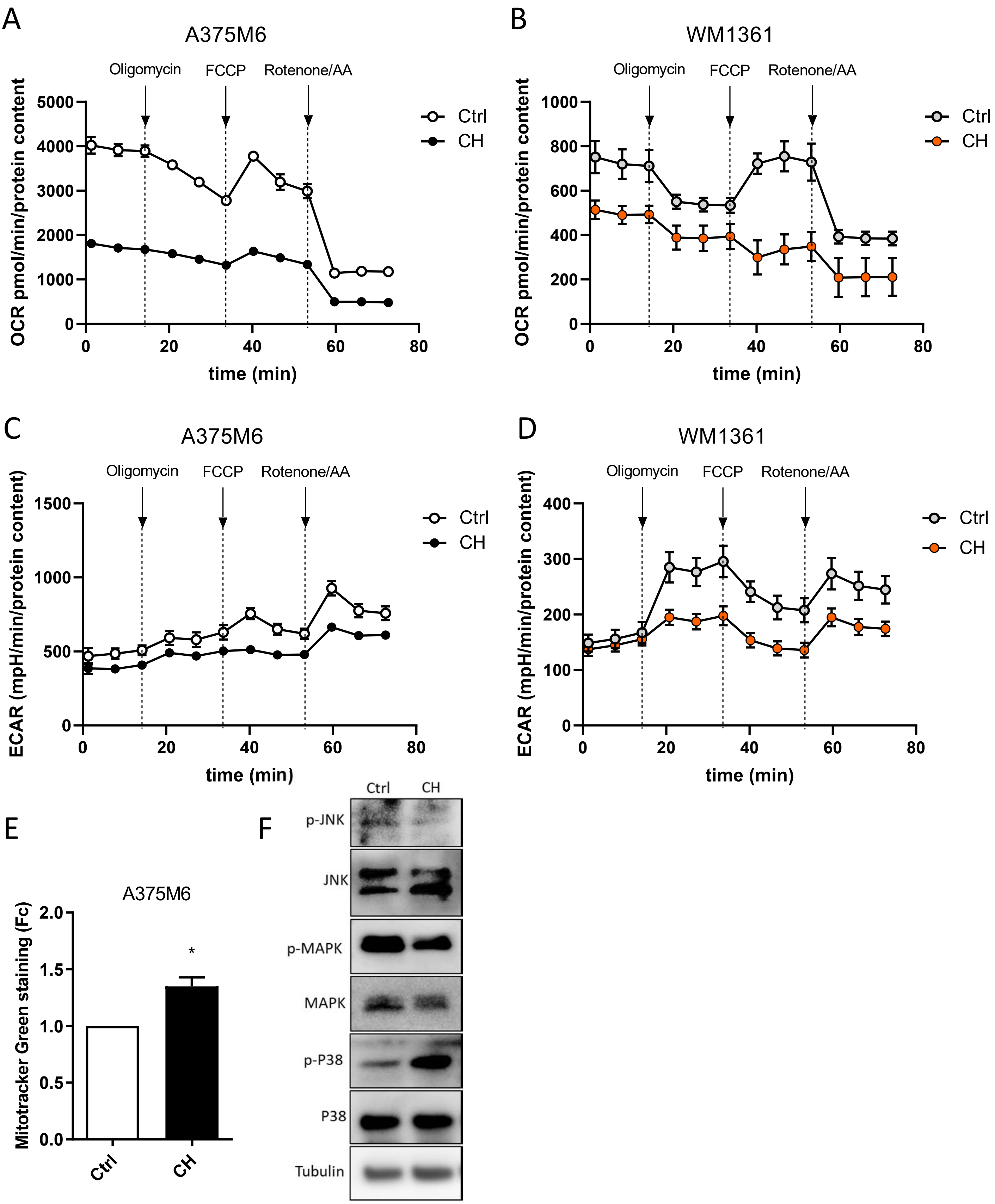

Supplementary Figure 3. (A, B) Oxygen consumption rate (OCR) measured in real time with Seahorse XFe96 Mito Stress Test analysis of A375M6 (A) and WM1361 (B) cells treated or not for 24 h with 10 μM CH or 5 μM CH respectively. The respiratory capacity was calculated based on the OCR after the administration of the ATP synthase inhibitor oligomycin, the proton uncoupler carbonilcyanide p-triflouromethoxyphenylhydrazone (FCCP), and the respiratory complex I inhibitor rotenone, together with the respiratory complex III inhibitor antimycin A. (C, D) Extracellular acidification rate (ECAR) measured in real time with Seahorse XFe96 Mito Stress Test analysis of A375M6 (C) and WM1361 (D) cells treated or not for 24 h with 10 μM CH or 5 μM CH respectively. (E) Mitochondrial mass of A375M6 cells following CH treatment. Mitochondrial mass was quantified by staining CH treated and non-treated A375M6 cells with MitoTracker Green probe and FACS analysis. (F) MAPK signaling pathway activation in A375M6 cells after CH treatment. Protein lysates from A375M6 melanoma cells, treated for 24 h with 10 μM CH, were analyzed by western blotting using specific antibodies. Anti-actin immunoblot was performed to assess equal loading for normalization. Images are representative of three independent experiments.
