# Supplementary material for: Claisened Hexafluoro Inhibits Metastatic Spreading of Amoeboid Melanoma Cells
Source: Cancers (Basel). 2021 Jul 15;13(14):3551. doi: 10.3390/cancers13143551 (PMC8305480; doi:10.3390/cancers13143551)
Supplement: Supplementary file 1 [file cancers-13-03551-s001.zip › cancers-1096282-supplementary-proof.pdf]

cancers

MDPI

Article

Supplementary Material: Claisened Hexafluoro Inhibits Metastatic Spreading of Amoeboid Melanoma Cells

Angela Leo [1], Erica Pranzini [1], Laura Pietrovito [1], Elisa Pardella [1], Matteo Parri [1], Paolo Cirri [1], Gennaro Bruno [2], Maura Calvani [2], Silvia Peppicelli [1], Eugenio Torre [1], Maiko Sasaki [3], Lily Yang [3], Lei Zhu [3], Paola Chiarugi [1], Giovanni Raugei [1], Jack L. Arbiser [3] and Maria Letizia Taddei [4,*]

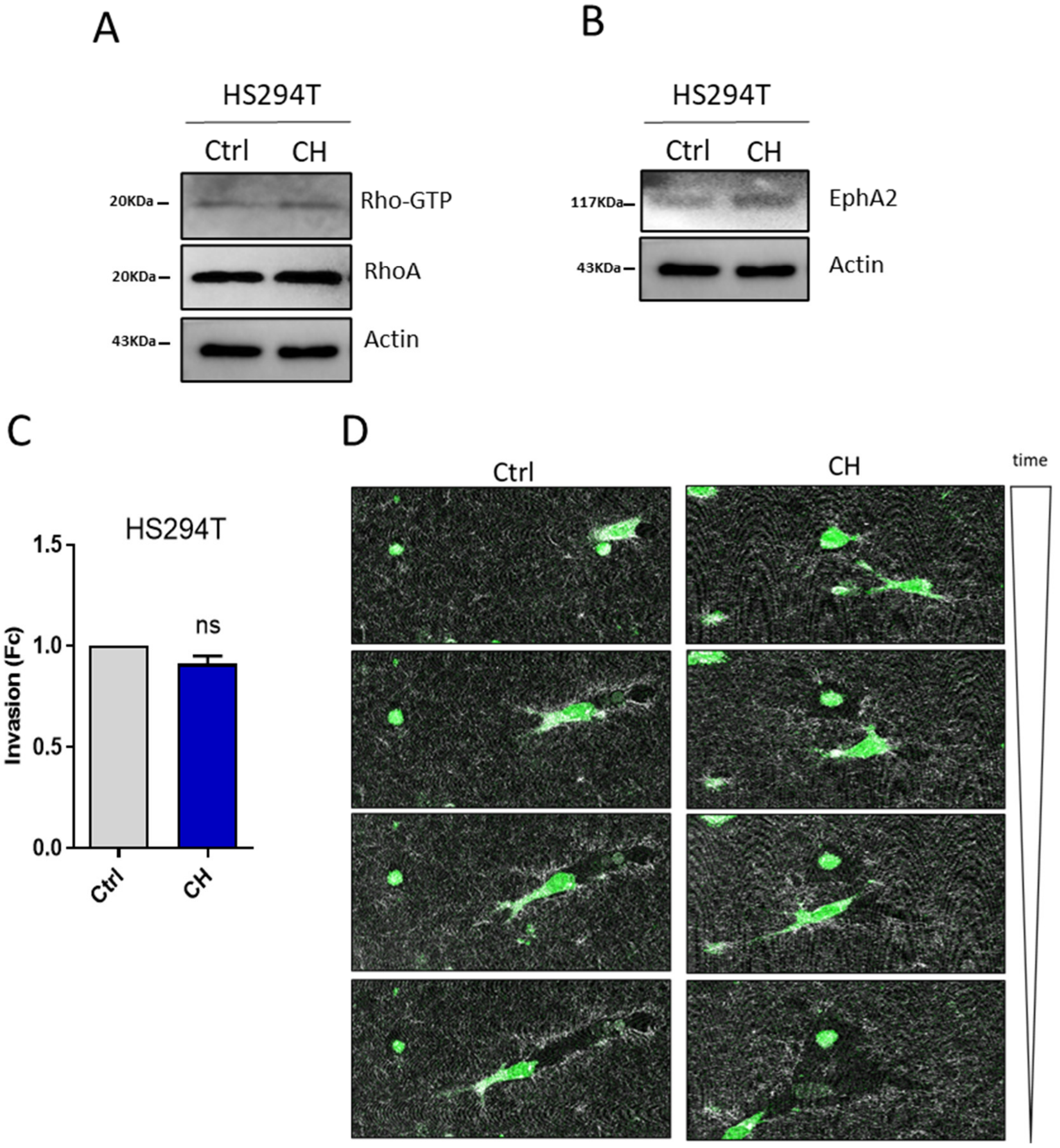

Figure S1. (A) Impact of CH treatment on RhoA activation in HS294T mesenchymal-like melanoma cells. RhoA activation was evaluated through pull-down assay on HS294T cells, following treatment with 10 µM CH for 24 h. Anti-total RhoA and anti-actin immunoblots were performed to assess equal protein loading. Images are representative of three independent experiments. (B) EphA2 protein levels in mesenchymal-like HS294T melanoma cell lines following CH treatment. Protein lysates from HS294T cells, treated for 24 h with 10 µM CH, were analyzed by western blotting using the anti-EphA2 antibody. Anti-actin immunoblot was performed to assess equal loading. Images are representative of three independent

experiments. (C) Invasion abilities of HS294T cells assessed by Boyden Chamber invasion assay. HS294T cells were treated for 24 h with 10 µM CH. Then, 5x104 cells were seeded in the upper compartment of a Boyden chamber coated with Matrigel and allowed to invade for 24 h toward complete medium (FBS 10%). Cell invasion was evaluated after Diff-Quick staining by counting cells in three randomly chosen fields. Data are reported as mean ± SEM from three independent experiments; t-test. (D) Migration of HS294T melanoma cells in three-dimensional collagen lattice. HS294T cells were treated with 10 µM CH for 24 h. CFSE labeled cells were incorporated into three-dimensional collagen I lattices and monitored by confocal fluorescence-reflection video microscopy for 16h. Melanoma cells are reported in green, whereas the back scatter signal of the collagen I is reported in white. Images are representative of three independent experiments. Scale bar: 10µM.

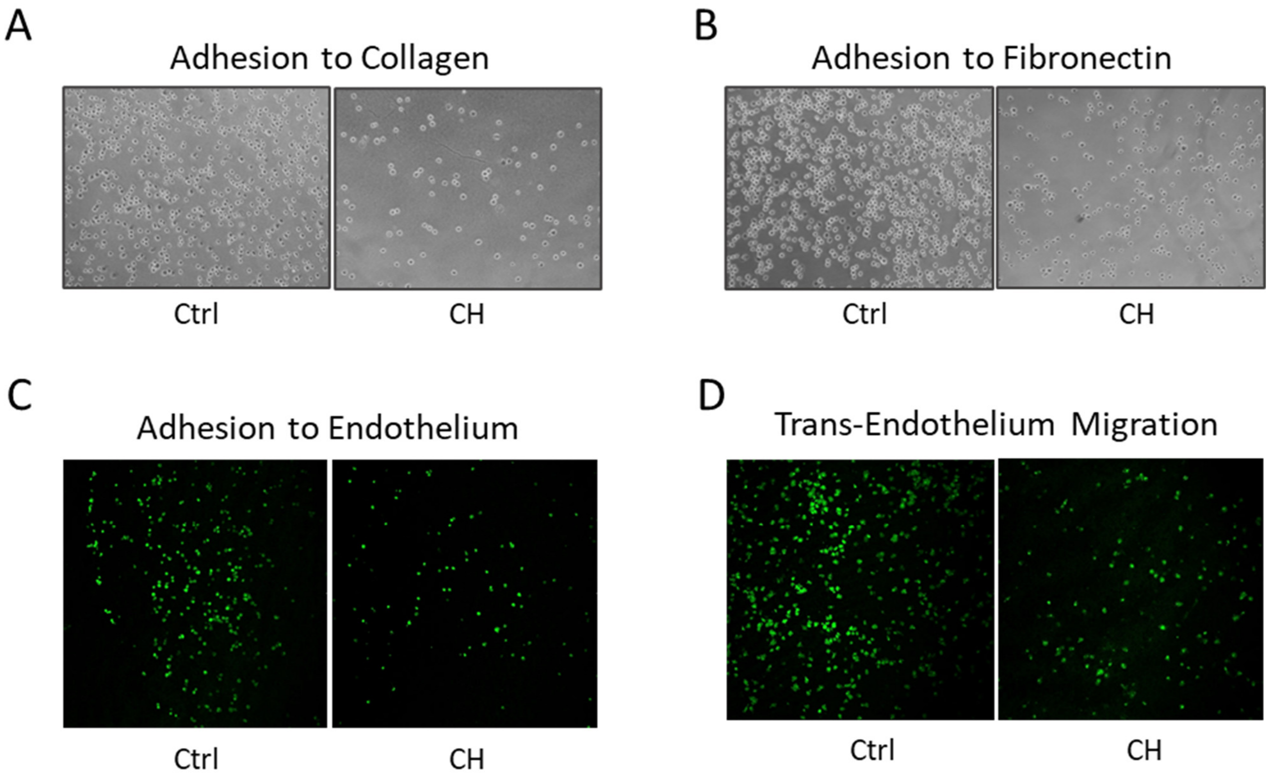

Figure S2. (A) A375M6 melanoma cell adhesion to collagen substrate. A375M6 cells, treated for 24 h with 10 µM CH, were let to adhere to culture plates previously coated with collagen for 10 min. Images are representative of five randomly chosen fields. Scale bar: 100µM (B) A375M6 melanoma cell adhesion to fibronectin substrate. A375M6 cells, treated for 24 h with 10 µM CH, were let to adhere to culture plates previously coated with fibronectin for 10 min. Images are representative of five randomly chosen fields. Scale bar: 100µM. (C) A375M6 melanoma cell adhesion to the endothelium. CFSE labeled A375M6 cells, treated with 10 µM CH for 24 h, were let to adhere for 30 min onto a monolayer of HUVEC cells. The adherent cells were visualized using an inverted fluorescent microscope and representative images are reported. Scale bar: 100µM. (D) A375M6 melanoma cell trans-endothelial migration ability. CFSE labeled A375M6 cells, treated with 10 µM CH for 24 h, were seeded onto a HUVEC monolayer in the upper compartment of a Boyden Chamber and let to migrate for 16 h toward complete medium (FBS 10%). Photos of fluorescent cells of five randomly chosen fields were acquired with inverted fluorescent microscope and representative images are reported. Scale bar: 100µM.

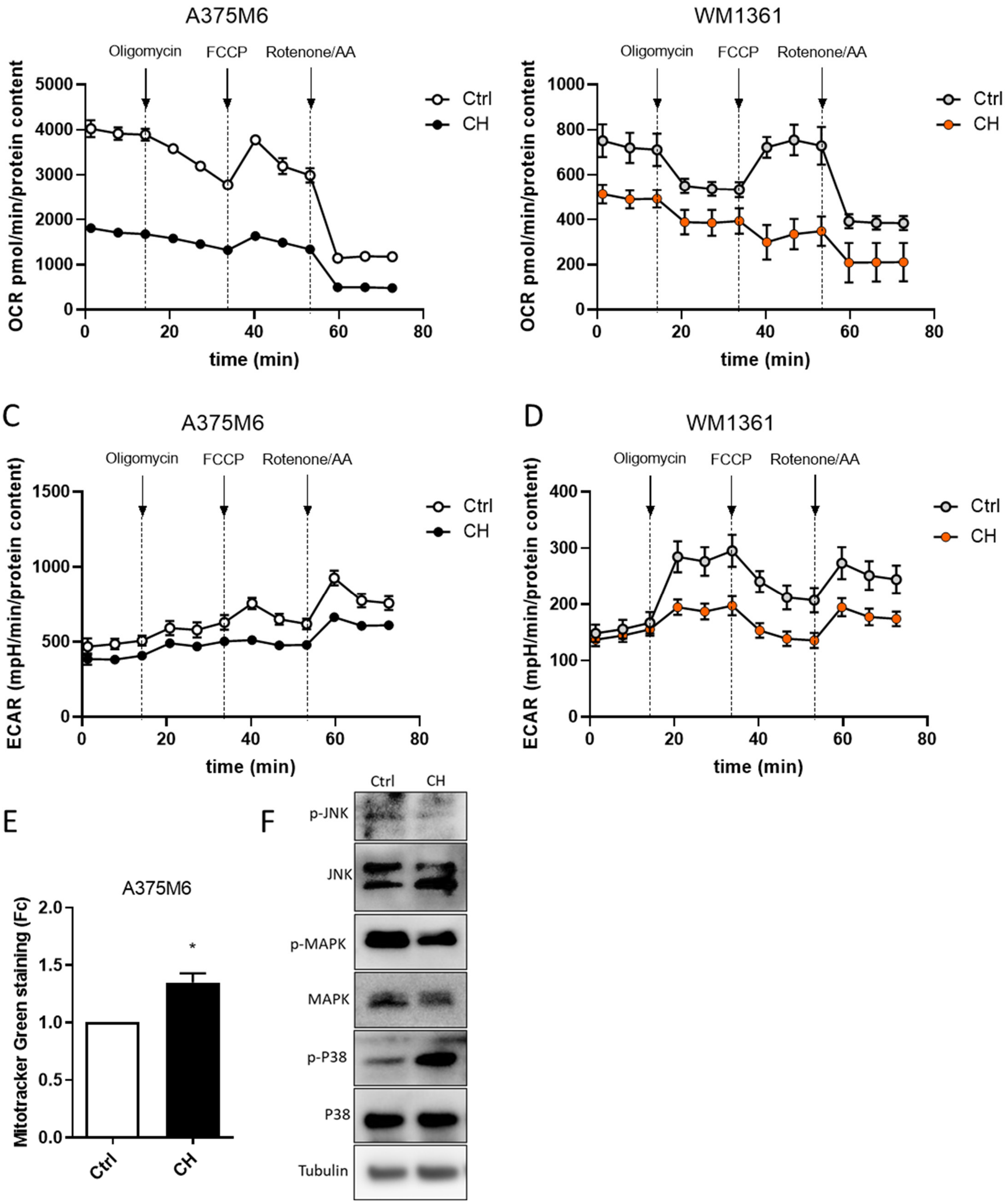

A375M6
WM1361
Oligomycin
FCCP
Rotenone/AA
Ctrl
CH
OCR pmol/min/protein content
time (min)
C
D
ECAR (mpH/min/protein content)
E
F
Mitotracker Green staining (Fc)
*
Ctrl
CH
p-JNK
JNK
p-MAPK
MAPK
p-P38
P38
Tubulin

Figure S3. (A, B) Oxygen consumption rate (OCR) measured in real time with Seahorse XFe96 Mito Stress Test analysis of A375M6 (A) and WM1361 (B) cells treated or not for 24 h with 10 μM CH or 5 μM CH respectively. The respiratory capacity was calculated based on the OCR after the administration of the ATP synthase inhibitor oligomycin, the proton uncoupler carbonilcyanide p-triflouromethoxyphenylhydrazone (FCCP), and the respiratory complex I inhibitor rotenone, together with the respiratory complex III inhibitor antimycin A. (C, D) Extracellular acidification rate (ECAR) measured in real time with Seahorse XFe96 Mito Stress Test analysis of A375M6 (C) and WM1361 (D) cells treated or not for 24 h with 10 μM CH or 5 μM CH respectively. (E) Mitochondrial mass of A375M6 cells following CH treatment. Mitochondrial mass was quantified by staining CH treated and non-treated A375M6 cells with MitoTracker Green probe and FACS analysis. (F) MAPK signaling pathway activation in A375M6 cells after CH treatment. Protein lysates from A375M6 melanoma cells, treated for 24 h with 10 μM CH, were analyzed by western blotting using specific antibodies. Anti-actin immunoblot was performed to assess equal loading for normalization. Images are representative of three independent experiments.
