# Supplementary figures and images for: Claisened Hexafluoro Inhibits Metastatic Spreading of Amoeboid Melanoma Cells
Source: Cancers (Basel). 2021 Jul 15;13(14):3551. doi: 10.3390/cancers13143551 (PMC8305480; doi:10.3390/cancers13143551)

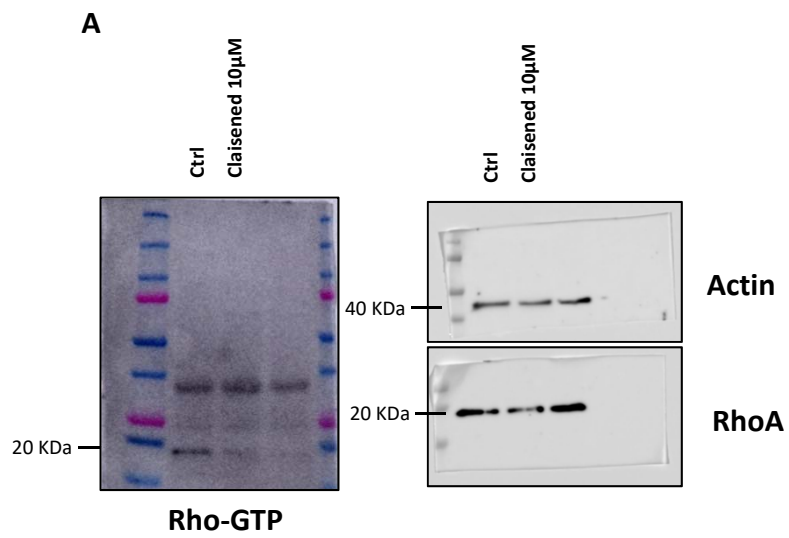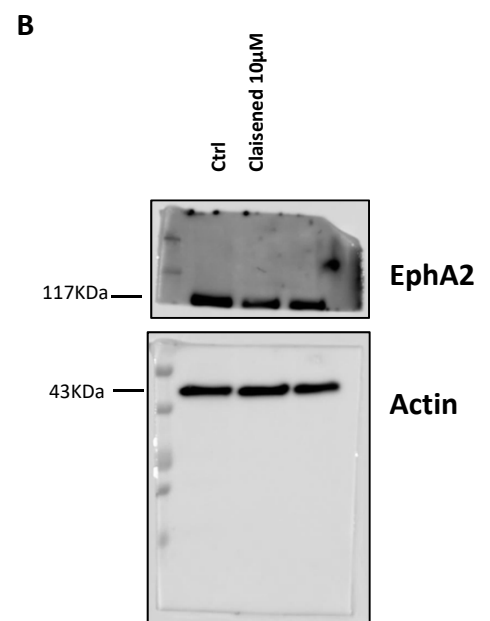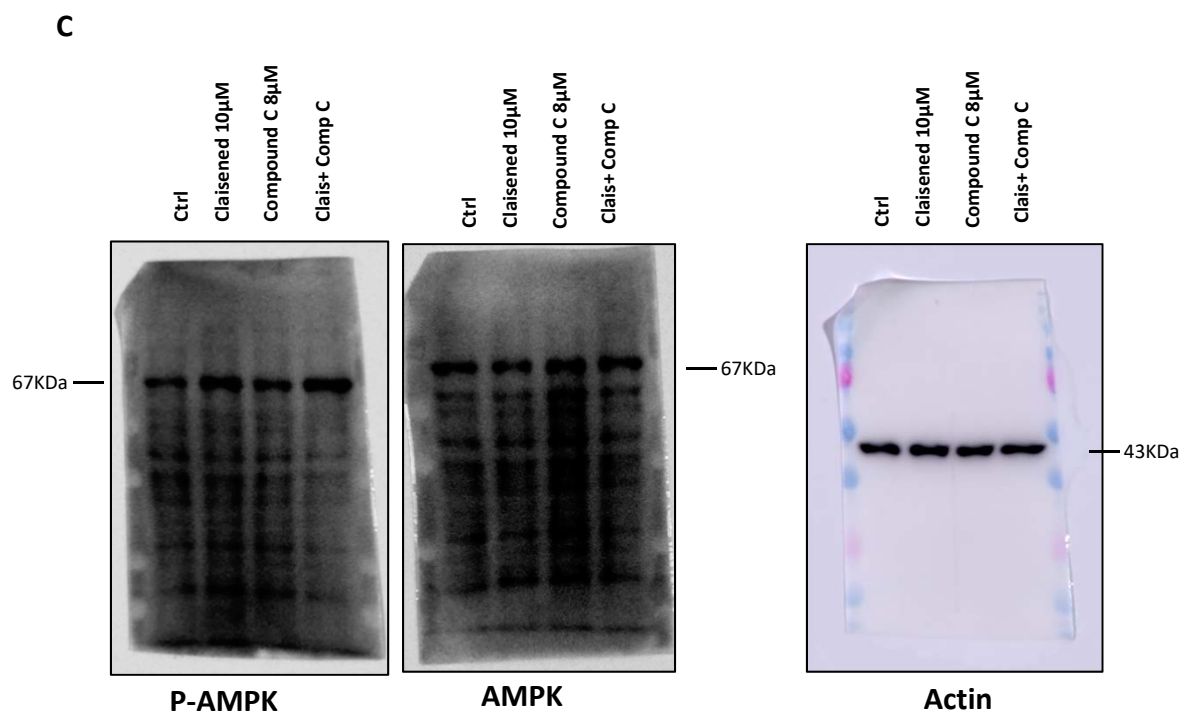

WM1361

Rho-GTP

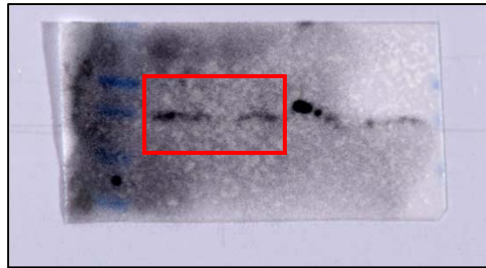

RhoA

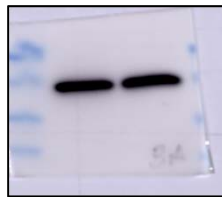

Actin for Rho

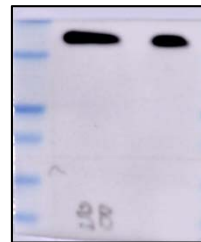

EphA

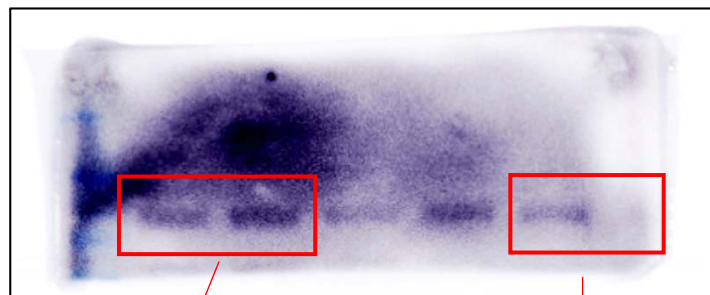

HS294T

WM1361

Actin for EphA in HS294T

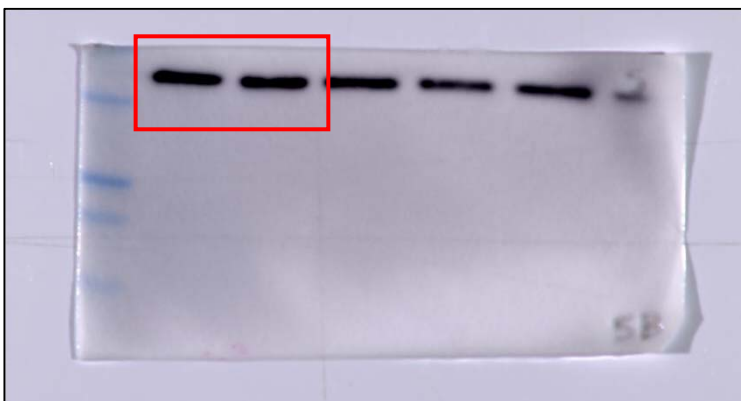

Actin for EphA in WM1361

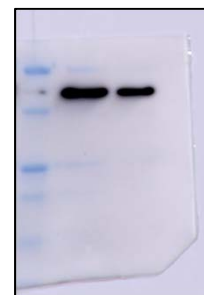

Supplement: Supplementary file 1 [file cancers-13-03551-s001.zip › Cancers-1096282-supplementary/Supplementary_Materials/Original_WB.pdf]
